# Relative vaccine effectiveness of mRNA COVID-19 boosters in people aged at least 75 years during the spring-summer (monovalent vaccine) and autumn-winter (bivalent vaccine) booster campaigns: a prospective test negative case–control study, United Kingdom, 2022

**DOI:** 10.2807/1560-7917.ES.2023.28.48.2300173

**Published:** 2023-11-30

**Authors:** Anastasia Chatzilena, Catherine Hyams, Rob Challen, Robin Marlow, Jade King, David Adegbite, Jane Kinney, Madeleine Clout, Nick Maskell, Jennifer Oliver, Adam Finn, Leon Danon, on behalf of The Avon CAP Research Group

**Affiliations:** 1 Engineering Mathematics, University of Bristol, Bristol, United Kingdom; 2 Bristol Vaccine Centre, Population Health Sciences, University of Bristol, Bristol, United Kingdom; 3 Population Health Sciences, University of Bristol, Bristol, United Kingdom; 4 Clinical Research and Imaging Centre, UHBW NHS Trust, Bristol, United Kingdom; 5 Academic Respiratory Unit, University of Bristol, Southmead Hospital, Bristol, United Kingdom; 6 Bristol Vaccine Centre, Cellular and Molecular Medicine and Population Health Sciences, University of Bristol, Bristol, United Kingdom; 7 The members of the group are listed under Acknowledgements; * These authors contributed equally to this work and share first/last authorship.

**Keywords:** COVID-19, SARS-CoV-2, respiratory infection, vaccination

## Abstract

**Background:**

Understanding the relative vaccine effectiveness (rVE) of new COVID-19 vaccine formulations against SARS-CoV-2 infection is a public health priority. A precise analysis of the rVE of monovalent and bivalent boosters given during the 2022 spring-summer and autumn-winter campaigns, respectively, in a defined population remains of interest.

**Aim:**

We assessed rVE against hospitalisation for the spring-summer (fourth vs third monovalent mRNA vaccine doses) and autumn-winter (fifth BA.1/ancestral bivalent vs fourth monovalent mRNA vaccine dose) boosters.

**Methods:**

We performed a prospective single-centre test-negative design case–control study in ≥ 75-year-old people hospitalised with COVID-19 or other acute respiratory disease. We conducted regression analyses controlling for age, sex, socioeconomic status, patient comorbidities, community SARS-CoV-2 prevalence, vaccine brand and time between baseline dose and hospitalisation.

**Results:**

We included 682 controls and 182 cases in the spring-summer booster analysis and 572 controls and 152 cases in the autumn-winter booster analysis. A monovalent mRNA COVID-19 vaccine as fourth dose showed 46.6% rVE (95% confidence interval (CI): 13.9–67.1) vs those not fully boosted. A bivalent mRNA COVID-19 vaccine as fifth dose had 46.7% rVE (95% CI: 18.0–65.1), compared with a fourth monovalent mRNA COVID-19 vaccine dose.

**Conclusions:**

Both fourth monovalent and fifth BA.1/ancestral mRNA bivalent COVID-19 vaccine doses demonstrated benefit as a booster in older adults. Bivalent mRNA boosters offered similar protection against hospitalisation with Omicron infection to monovalent mRNA boosters given earlier in the year. These findings support immunisation programmes in several European countries that advised the use of BA.1/ancestral bivalent booster doses.

**Key public health message**

**What did you want to address in this study?**

Understanding the additional protection offered by COVID-19 boosters against SARS-CoV-2 infection is an urgent public health priority. We therefore compared the protection against hospitalisation provided by the two vaccines used in the booster campaigns in the United Kingdom in 2022: the original monovalent mRNA vaccine versus the bivalent mRNA booster targeting both the original virus strain and the Omicron BA.1 variant.

**What have we learnt from this study?**

Both vaccine formulations demonstrated benefit as a booster in people ≥ 75 years. The bivalent COVID-19 boosters distributed during the 2022 autumn-winter campaign augmented the protection against hospitalisation after infection with the Omicron variant at a level equivalent to the original monovalent boosters offered during the 2022 spring-summer campaign.

**What are the implications of your findings for public health?**

Our findings support the booster immunisation programmes implemented in several European countries, that advised the use of BA.1/ancestral mRNA bivalent booster doses in individuals at high risk of severe COVID-19.

## Introduction

Following the emergence of wild-type severe acute respiratory syndrome coronavirus 2 (SARS-CoV-2) and circulation of antigenically distinct variants, large-scale vaccination programmes were implemented to reduce overall COVID-19 morbidity and mortality. In the United Kingdom (UK), several COVID-19 vaccines received rapid regulatory authorisation: the vaccines used initially were the monovalent mRNA vaccine Cominarty (BNT162b2; Pfizer-BioNTech) and the Vaxzevria replication-deficient simian adenovirus vector vaccine (ChAdOx1; AstraZeneca), with Spikevax (mRNA-1273 vaccine; Moderna) approved a few months later. These three COVID-19 vaccines were used in the primary campaign in the UK which began in December 2020, using initially an extended interval between first and second doses equal to 12 weeks, to prioritise first dose administration. The mRNA vaccines were offered as boosters 6 months after completion of the primary course, from September 2021 for adults aged ≥ 50 years and those in clinical risk groups, extending to all adults in November 2021. A fourth dose of an mRNA vaccine was offered from March 2022 and prioritised the most vulnerable people: all adults aged ≥ 75 years and those in clinical risk groups [1]. Immunosuppressed individuals had already received a third dose as part of the priming vaccinations in early 2021, so that for them, the autumn 2021 and spring 2022 boosters were generally their fourth and fifth doses, respectively. The COV-Boost study indicated that a fourth-dose COVID-19 mRNA vaccination boosts immune responses [2], and an observational study showed three- or four-dose vaccine effectiveness (VE) against hospitalisation of 60.9–62.1% against the BA.4 or BA.5 variants which emerged during spring 2022 and 50.1% against BA.2 when compared with two doses received ≥ 25 weeks earlier [3]. These initial COVID-19 vaccines were developed against wild-type virus and provided substantial protection against infection, hospitalisation, severe disease and death [4-8]. However, it has been observed that VE can be eroded progressively both by waning of immune protection over time and emergence of SARS-CoV-2 variants of concern (VOC) (Alpha, Delta, Omicron and its subvariants) which show immune escape [9-12].

In summer 2022, the UK Medicines and Healthcare products Regulatory Agency (MHRA) approved two new bivalent booster vaccines which were developed in response to concerns about such viral evolution and escape. The Spikevax bivalent Original/Omicron vaccine (Moderna) was approved on 15 August 2022, followed quickly by the Comirnaty Original/Omicron BA.1 bivalent vaccine (Pfizer-BioNTech) approval on 3 September 2022 [13,14] and they were distributed during autumn 2022, being the fifth dose offered in the UK. The bivalent Spikevax vaccine contains 25 μg of mRNA coding for the spike protein of the ancestral strain and 25 μg of mRNA against Omicron (BA.1), and the Comirnaty vaccine contains 15 μg of mRNA directed against the ancestral strain and 15 μg of mRNA against Omicron (BA.1). Early immunogenicity studies suggest that bivalent mRNA boosters induce similar or higher neutralising antibody levels against Omicron subvariants and other VOCs compared with monovalent mRNA boosters [15-19].

As of the end of February 2023, SARS-CoV-2 infection incidence remains high [20], while determining whether patients who test SARS-CoV-2-positive have COVID-19 has become increasingly challenging using studies relying on data linkage methodology. In addition, comparisons between vaccinated individuals and those who have not received any COVID-19 vaccine dose cannot be performed, as 78.2% of the adult population in the UK have received at least two doses or had prior exposure to SARS-CoV-2: thus even unvaccinated individuals have some immunity to SARS-CoV-2. There remains limited evidence of bivalent vaccines’ clinical effectiveness compared with monovalent formulations because the different vaccine rollout timings make a direct comparison of the vaccines impossible [10,15,21].​ Acknowledging this constraint, we undertook a test-negative design case–control study comparing SARS-CoV-2-positive and -negative patients with acute lower respiratory tract disease (aLRTD), implementing two separate analyses to assess the protection against SARS-CoV-2 hospitalisation provided by an additional monovalent or BA.1/ancestral bivalent mRNA vaccine dose relative to those who had not received the respective doses. We focused on ≥ 75-year-old patients who were the main target group in the spring 2022 booster programme. Given the different rollout timings of the two vaccine formulations, the analyses refer to two distinct study time periods with different subvariants circulating.

## Methods

### Study design and conduct

We estimated the relative vaccine effectiveness (rVE) of monovalent and bivalent mRNA vaccines against COVID-19 hospitalisation in Bristol, within the study population consisting of adults admitted with lower respiratory tract infections to North Bristol and University Hospitals Bristol and Weston NHS Trusts (AvonCAP study registration number: ISRCTN17354061) between 4 April 2022 and 30 July 2022 (the period following the initiation of distribution of the fourth dose of monovalent mRNA vaccines) and between 21 September 2022 and 23 January 2023 inclusive (the period following the initiation of distribution of bivalent mRNA vaccines), and tested for SARS-CoV-2 infection. During the first study period, BA.4, BA.2 and BA.5 were the main SARS-CoV-2 Omicron sub-lineages identified in COVID-19 cases in England, while the dominant sub-lineages identified during the second study period were BA.5, BA.4.6, BQ.1, CH1.1, XBB recombinant lineage and its mutation XBB.1.5; we provide additional details on the number of admissions by vaccination status and the prevalence of different SARS-CoV-2 variants over time in Supplementary Figure S1 [22]. The study population consisted of patients with signs/symptoms of respiratory infection, aged ≥ 18 years at the time of hospitalisation [23]. We identified eligible cases and controls from the medical admission list and collected data from medical records using REDCap software [24]. All data collection was undertaken by individuals not involved in analysis and unaware of the results, following the same procedures for both cases and controls. Vaccination records for every study participant were obtained from linked hospital and GP records, including vaccine brand and date of administration, with data collection performed by individuals unaware of the participants’ SARS-CoV-2 test results [25].

### Case definition and exclusions

We included patients with two or more signs of acute respiratory disease (cough, fever, dyspnoea, tachypnoea, increased/discoloured sputum expectoration, pleurisy, clinical or radiological findings suggestive of acute disease) or a confirmed clinical/radiological diagnosis of aLRTD [25]. Patients hospitalised with aLRTD and positive SARS-CoV-2 test at admission using the UK Health Security Agency (UKHSA) diagnostic assay in use at the time were classified as cases; those with aLRTD and negative SARS-CoV-2 result were classified as controls. Eligible controls could have multiple hospitalisations, provided subsequent admissions were > 7 days following previous discharge. We included only the first COVID-19 admission for each case.

We excluded patients whose admission date was > 10 days after symptom onset date (to avoid including potentially false-negative admission SARS-CoV-2 tests), and those with a confirmed previous SARS-CoV-2 infection based on any positive test result that could be found in a local and/or national database of clinical care records, including linkage through the UKHSA national testing system. Patients who had received two vaccine doses or fewer at the time of admission were also excluded; inclusion and exclusion criteria are listed in Supplementary Table S1.

In order to make a side-by-side evaluation of the effectiveness of the different booster vaccine formulations, we restricted both analyses to individuals aged ≥ 75 years, since the Joint Committee on Vaccination and Immunisation (JCVI) advised targeting COVID-19 booster vaccines during spring and summer towards those at highest risk of severe disease, those aged ≥ 75 years and residents in long-term care facilities (LTCFs) [1], while the offer was extended in autumn and winter 2022, including those aged ≥ 50 years and frontline health and social care workers [26].

### Exposure definition

This analysis aimed to measure the protection offered by an additional dose of monovalent (original ‘wild-type’ mRNA vaccine, Comirnaty or Spikevax) and bivalent (original ‘wild-type’/Omicron BA.1 mRNA vaccine, Comirnaty or Spikevax) vaccine within 3 months after vaccination, each compared with people who had not received the respective boosters, side by side, during SARS-CoV-2 Omicron variant dominance. The spring-summer monovalent booster analysis (admissions 4 April–30 July 2022) compared the fourth dose of monovalent vaccine given as a booster (21 March–7 August 2022) in the UK, to the third dose of monovalent vaccine during autumn-winter 2021 (16 September 2021–14 February 2022). By the end of that period, the vaccine uptake in the UK in people ≥ 75 years was 74.3% for the spring 2022 booster and 93.5% for three doses (compared with a median vaccine uptake of 13.6% and 84.2%, respectively, in EU/EEA countries in over 60-year-olds based on available data [27]). The autumn-winter bivalent booster analysis (admissions 21 September 2022–23 January 2023) compared the fifth dose of vaccine, with the bivalent formulation given as a booster (7 September 2022–12 February 2023) to the fourth dose of monovalent vaccine in spring-summer 2022 (21 March–7 August 2022). The vaccine uptake in individuals aged ≥ 75 years by the end of this study period was 83.5% for autumn 2022 booster and 74.9% for spring 2022 booster (compared with a median vaccine uptake of 2.2% and 35.1%, respectively, in EU/EEA countries in over 60-year-olds based on available data [27]).

For the spring-summer monovalent booster analysis, individuals were defined as boosted with a monovalent vaccine if they had received three doses of any monovalent vaccine combination and a fourth dose of monovalent vaccine during the spring-summer 2022 vaccination programme, and no more than 3 months before their admission to hospital; they were defined as not fully boosted if they had received only two doses of any vaccine combination followed by a third dose of monovalent vaccine during autumn-winter 2021. For the autumn-winter bivalent booster analysis, individuals were defined as boosted with a BA.1/ancestral bivalent vaccine if they had received four doses of any vaccine combination plus a fifth bivalent dose during the spring-summer 2022 vaccination programme and no more than 3 months before their admission; they were defined as not fully boosted if they had received three doses of any vaccine combination plus a fourth monovalent dose during autumn-winter 2022. In both analyses, we defined as immunised those who had received their most recent dose > 7 days before symptom onset; for in- and exclusion criteria see Supplementary Table S1.

**Figure 1:**
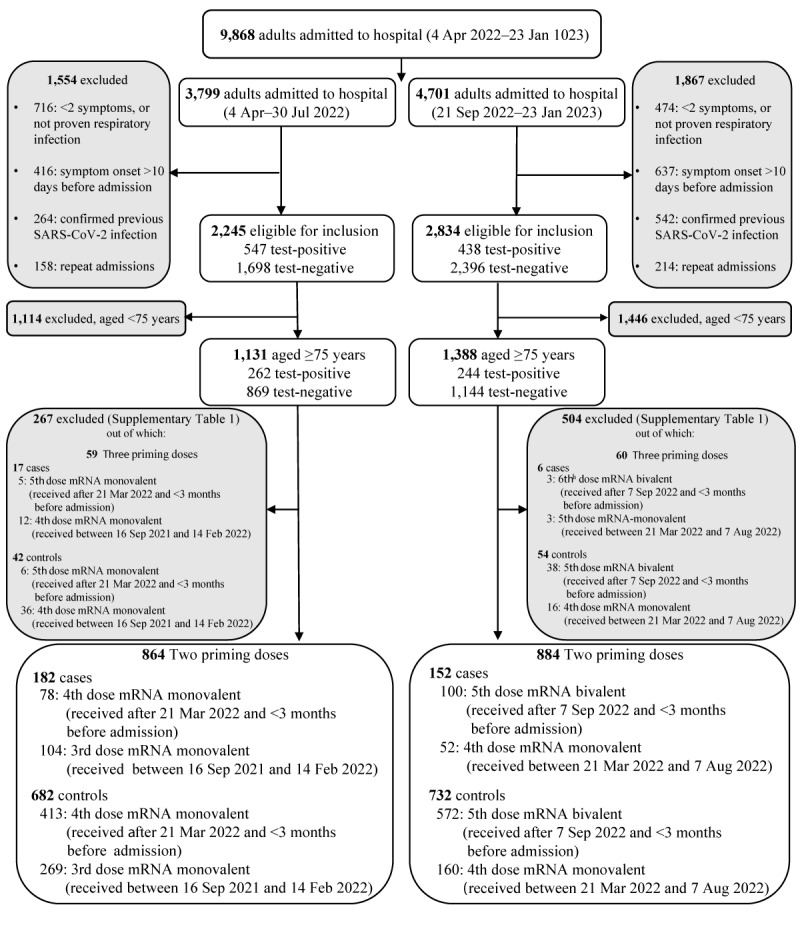
Study flow diagram, inclusion and exclusion criteria, case–control study on COVID-19 booster vaccine effectiveness, United Kingdom, April 2022–January 2023 (n = 9,868)

**Figure 2:**
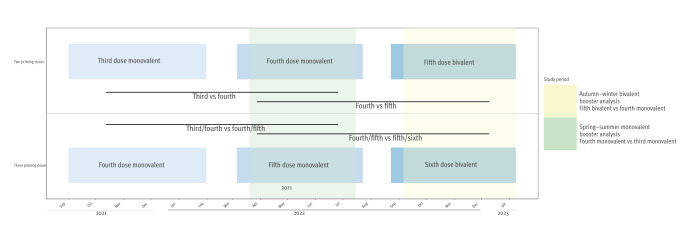
Study timeline, case–control study on COVID-19 booster vaccine effectiveness, United Kingdom, 2022

Individuals who had received a third vaccine dose in autumn-winter 2021, a fourth dose in spring-summer 2022 and a fifth dose in autumn-winter 2022, are those who had received two doses as the primary vaccination regimen before and during spring-summer 2021. However, individuals with severe immunosuppression around the time of their first or second vaccine doses were offered an additional primary dose (third dose) before any booster doses. As a result, they were offered a fourth vaccine dose in autumn-winter 2021, a fifth dose in spring-summer 2022 and a sixth dose in autumn-winter 2022 (Figures 1 and 2). Additional detail on the number of admissions by vaccination group over time is appended in Supplementary Figure S1. Since this population almost exclusively comprised of immunosuppressed individuals, we performed additional sensitivity analyses including those individuals who had received three doses as their primary vaccination regimen in both comparisons.

The left panel describes the spring-summer monovalent booster analysis; of the 182 SARS-CoV-2-positive individuals, 78 were vaccinated with fourth-dose monovalent mRNA vaccine and 104 were vaccinated with third-dose monovalent mRNA vaccine. Of the 682 SARS-CoV-2-negative individuals admitted, 413 were vaccinated with fourth-dose monovalent mRNA vaccine and 269 were vaccinated with third-dose monovalent mRNA vaccine.

The right panel describes the autumn-winter bivalent booster analysis; of the 152 SARS-CoV-2-positive individuals, 100 were vaccinated with fifth-dose bivalent mRNA vaccine and 52 were vaccinated with fourth-dose monovalent mRNA vaccine. Of the 732 SARS-CoV-2-negative individuals admitted, 572 were vaccinated with fifth-dose bivalent mRNA vaccine and 160 were vaccinated with fourth dose monovalent mRNA vaccine.

### Outcomes

We assessed the additional protection provided by a fourth dose of mRNA monovalent vaccine and a fifth dose of BA.1/ancestral bivalent vaccine as boosters against the primary endpoint of hospital admission with a positive SARS-CoV-2 test at admission and either a clinical or radiological aLRTD diagnosis or aLRTD signs/symptoms compared with the protection provided by three or four doses of the monovalent formulations of the vaccines, respectively.

### Statistical analysis

Demographic and clinical characteristics, and other factors that may affect the exposure (vaccination status) or outcome (hospital admission), were compared between cases and controls for both comparisons, between those boosted with a monovalent vaccine and not fully boosted, and between those boosted with a bivalent vaccine and not fully boosted, using Fisher’s exact tests (categorical variables), two-sided Kolmogorov–Smirnov tests (continuous variables) and Wilcoxon rank-sum tests (score variables).

Under test-negative design assumptions, we estimated the odds ratio (OR) of testing SARS-CoV-2-positive among patients boosted with a monovalent vaccine vs those not fully boosted (rOR) and defined rVE as (1 − rOR) × 100. Similarly, we estimated rVE of bivalent booster, comparing the odds of testing positive for SARS-CoV-2 among patients boosted with a bivalent vaccine vs those not fully boosted. This was done using univariable logistic regression (univariable logistic regression model). Differences in the timing of the third/fourth dose and roll-out timing of different vaccine brands could introduce unobserved biases, confounding results in both comparisons. To mitigate these, we performed multivariable logistic regression analyses adjusting for time between ‘baseline vaccine dose’ (third dose for the spring-summer monovalent booster and fourth dose for the autumn-winter bivalent booster analysis) and admission (in days), vaccine brand (binary variable) age, sex (binary variable), index of multiple deprivations (IMD) decile rank and Charlson comorbidity index (CCI) (continuous variable), LTCF residency status, presence of pre-existing respiratory disease, and community SARS-CoV-2 prevalence lagged by time interval between infection and hospitalisation, assumed to be 8 days (multivariable logistic regression model).

We also conducted sensitivity analyses, matching cases and controls using propensity score balancing using logistic regression to define propensity score, and nearest neighbour matching. Matching variables included age, sex, CCI and IMD, LTCF residency status and presence of pre-existing respiratory disease, and likelihood of vaccine receipt (matched conditional logistic regression model). Matching by elapsed time since baseline vaccine dose/brand was not performed to avoid introducing bias [28]. Time since baseline vaccine dose/ brand is affected by dose of last vaccine received; each booster was deployed ≥ 4 months after the previous COVID-19 booster programme (Figure 2), with different programmes using different proportions of each vaccine brand. As an additional sensitivity analysis, we included individuals who had received three doses as primary vaccination regimen, adjusting for the number of primary doses (binary variable), using the same methods for both comparisons.

Statistical analyses were performed with R version 4.0.2 (R Foundation). Missing data were limited to the IMD variable and accounted for < 1%; no imputation was performed; all analyses only included participants with complete data. Statistical significance was defined using a two-sided significance level of α = 0.05.

## Results

During the periods evaluated, 9,868 adult aLRTD hospitalisations occurred in Bristol, UK, while the Omicron variant was dominant [22,29,30]. In the spring-summer booster, 864 admissions of ≥ 75-year-old patients hospitalised with SARS-CoV-2 aLRTD were eligible for this analysis: median patient age was 85 years (interquartile range (IQR): 80–89), 403 individuals (46%) were male, median CCI was 5 (IQR: 4–6). No significant differences were observed in patient age and sex between SARS-CoV-2-positive and -negative aLRTD patients, while there was a statistically significant difference in their ethnicity, LTCF residency status, smoking and presence of pre-existing respiratory disease and chronic obstructive pulmonary disease (Figure 1, Table 1). In the autumn-winter booster, 884 admissions of ≥ 75-year-old patients were eligible with no significant differences in age, sex and LTCF residency status between SARS-CoV-2-positive and -negative aLRTD patients, while differences in ethnicity, smoking and dementia status were statistically significant (Figure 1, Table 2). In both comparisons, there were no significant differences in patient demographics and health status between vaccination groups. For details on patients’ characteristics by vaccination group see Supplementary Tables S2 and S3.

**Table 1:** Admission characteristics of study participants ≥ 75 years, fourth dose relative to third dose monovalent mRNA vaccines, admitted between 4 April and 30 July 2022 (n = 864)

Characteristic	Cases SARS-CoV-2-positive (n = 182)		Controls SARS-CoV-2-negative (n = 682)		p value
	n	%	n	%	
Vaccination status^a^					
4th mRNA monovalent	78	43	413	61	< 0.001
3rd mRNA monovalent	104	57	269	39	
Vaccine brand**^b^**					
SpikeVax (Moderna)	53	29	271	40	0.010
Comirnaty (Pfizer-BioNTech)	129	71	411	60	
Median days since vaccination (IQR)					
Time since 3rd dose	191 (165–235)		205 (174–237)		0.028
Time since 2nd dose	402 (368–444)		412 (382–443)		0.002
Time since last dose	140 (65–175)		72 (35–165)		< 0.001
Months since last dose					
≤ 3months	79	43	419	61	< 0.001
> 3months	103	57	263	39	
Median age in years (IQR)	85 (79–90)		84 (80–89)		0.4
Sex					
Male	96	53	319	47	0.2
Female	86	47	363	53	
LTCF resident	13	7.1	93	14	0.016
Ethnicity					
White British	147	91	538	96	0.043
Other	14	8.7	24	4.3	
Unknown	21	NI	120	NI	
Median IMD (IQR)	5 (4–8)		5 (4–8)		> 0.9
Unknown	3	NI	7	NI	
Smoking					
Current	12	6.8	33	5.1	0.033
Ex-smoker	94	53	414	64	
Non-smoker	70	40	198	31	
Unknown	6	NI	37	NI	
Comorbidity scores					
Rockwood frailty scale					
1–4	44	34	129	30	0.4
5–9	85	66	304	70	
Unknown	53	NI	249	NI	
Median CCI (IQR)	5 (4–6)		5 (4–6)		0.9
Respiratory					
Any**^c^**	63	35	306	45	0.014
COPD	39	21	228	33	0.002
Asthma	16	8.8	69	10	0.7
Other**^d^**	16	8.8	70	10	0.7
Cardiovascular					
Any	99	54	387	57	0.6
IHD	30	16	125	18	0.7
AF	59	32	218	32	> 0.9
CCF	42	23	179	26	0.4
Diabetes					
Any	46	25	142	21	0.2
Type 1	0	0	0	0	
Type 2	46	100	142	100	
Neurological					
Dementia	24	13	93	14	> 0.9
Cognitive impairment	15	8.2	37	5.4	0.2
CVA	18	9.9	73	11	0.9
TIA	14	7.7	67	9.8	0.5
Other neurological disease**^e^**	11	6.0	32	4.7	0.4
Immunodeficiency					
CTD	19	10	67	9.8	0.8
HIV	0	0	0	0	NA
Other immunodeficiency	15	8.2	52	7.6	0.8
Oncology					
Solid organ cancer	17	9.3	70	10	0.8
Haematological malignancy	3	1.6	7	1.0	0.4
Renal disease**^f^**					
None	105	58	414	61	0.2
Mild	64	35	241	35	
Moderate/severe	13	7.1	27	4.0	

AF: atrial fibrillation; CCF: congestive cardiac failure; CCI: Charlson comorbidity index; COPD: chronic obstructive pulmonary disease; CKD: chronic kidney disease; CTD: connective tissue disease; CVA: cerebrovascular accident; IHD: ischaemic heart disease; IMD: index of multiple deprivation; IQR: interquartile range; LTCF: long-term care facility; NA: not applicable; NI: not included in denominator; TIA: transient ischaemic attack.

**^a^** Vaccinated individuals who received a fourth dose of monovalent mRNA vaccine after 21 March 2022 and less than 3 months before admission or a third dose of monovalent mRNA vaccine between 16 September 2021 and 14 February 2022.

**^b^** Refers to vaccine brand of the last dose received (fourth or third). Prior to last dose, any vaccine combination is considered.

**^c^** Includes any chronic respiratory condition on admission to hospital, such as COPD, asthma, bronchiectasis, pulmonary fibrosis or a rare lung disease.

**^d^** Includes bronchiectasis, pulmonary fibrosis and other rare chronic respiratory conditions.

**^e^** Includes Parkinson’s disease, Huntingdon’s disease and other chronic neurological conditions.

**^f^** Mild is CKD stage 1–3; moderate or severe is CKD stage 4–5, end-stage renal failure or dialysis dependence.

**Table 2:** Admission characteristics of study participants ≥ 75 years, fifth dose BA.1/ancestral mRNA bivalent relative to fourth dose monovalent vaccines, admitted between 21 September 2022 and 23 January 2023 (n = 884)

Characteristic	Cases SARS-CoV-2-positive (n = 152)		Controls SARS-CoV-2-negative (n = 732)		p value
	n	%	n	%	
Vaccination status^a^					
5th mRNA bivalent	100	66	572	78	0.002
4th mRNA monovalent	52	34	160	22	
Vaccine brand^b^					
SpikeVax (Moderna)	93	61	481	66	0.3
Comirnaty (Pfizer-BioNTech)	59	39	251	34	
Median days since vaccination (IQR)					
Time since 4th dose	205 (169–238)		210 (179–244)		0.4
Time since 3rd dose	386 (353–418)		394 (362–421)		0.3
Time since 2nd dose	595 (552–620)		601 (569–625)		0.1
Time since last dose	75 (52–145)		67 (40–97)		0.023
Months since last dose					
≤ 3 months	101	66	578	79	0.001
> 3 months	51	34	154	21	
Median age in years (IQR)	85 (81–89)		85 (80–89)		0.6
Sex					
Male	78	51	325	44	0.13
Female	74	49	407	56	
LTCF resident	18 (12%)		87 (12%)		> 0.9
Ethnicity					
White British	109	96	583	98	0.3
Other	4	3.5	13	2.2	
Unknown	39		136		
Median IMD (IQR)	7 (4–9)		6 (4–9)		0.4
Unknown	2	NI	9	NI	
Smoking					
Current	2	1.4	48	6.9	0.003
Ex-smoker	84	60	454	65	
Non-smoker	54	39	195	28	
Unknown	12	NI	35	NI	
Comorbidity scores					
Rockwood frailty scale					
1–4	45	38	269	46	0.13
5–9	73	62	318	54	
Unknown	34	NI	145	NI	
Median CCI (IQR)	5 (4–6)		5 (4–6)		> 0.9
Respiratory					
Any^c^	59	39	322	44	0.3
COPD	37	24	219	30	0.2
Asthma	18	12	89	12	> 0.9
Other^d^	9	5.9	73	10	0.13
Cardiovascular					
Any	85	56	408	56	> 0.9
IHD	30	20	111	15	0.2
AF	49	32	240	33	> 0.9
CCF	31	20	179	24	0.3
Diabetes					
Any	26	17	139	19	0.6
Type 1	0	0	1	0.7	> 0.9
Type 2	26	100	138	99	
Neurological					
Dementia	26	17	81	11	0.041
Cognitive impairment	18	12	56	7.7	0.11
CVA	20	13	65	8.9	0.13
TIA	18	12	64	8.7	0.2
Other neurological disease^e^	11	7.2	34	4.6	0.2
Immunodeficiency					
CTD	16	11	63	8.6	0.4
HIV	0	0	1	0.1	> 0.9
Other immunodeficiency	20	13	103	14	0.9
Oncology					
Solid organ cancer	12	7.9	65	8.9	0.9
Haematological malignancy	4	2.6	21	2.9	> 0.9
Renal disease^f^					
None	88	58	386	53	0.5
Mild	59	39	312	43	
Moderate/severe	5	3.3	34	4.6	

AF: atrial fibrillation; CCF: congestive cardiac failure; CCI: Charlson comorbidity index; COPD: chronic obstructive pulmonary disease; CKD: chronic kidney disease; CTD: connective tissue disease; CVA: cerebrovascular accident; IHD: ischaemic heart disease; IMD: Index of multiple deprivation; IQR: interquartile range; LTCF: long-term care facility; NI: not included in denominator; TIA: transient ischaemic attack.

^a^ Vaccinated individuals who received a fifth dose of BA.1/ancestral mRNA bivalent mRNA vaccine after 7 September 2022 and less than 3 months before admission or a fourth dose of monovalent mRNA vaccine between 21 March and 7 August 2022.

^b^ Refers to vaccine brand of the last dose received (fifth or fourth dose). Prior to last dose, any vaccine combination is considered.

^c^ Includes any chronic respiratory condition on admission to hospital, such as COPD, asthma, bronchiectasis, pulmonary fibrosis or a rare lung disease.

^d^ Includes bronchiectasis, pulmonary fibrosis and other chronic respiratory conditions.

^e^ Includes Parkinson’s disease, Huntingdon’s disease and other chronic neurological conditions.

^f^ Mild is CKD stage 1–3; moderate or severe is CKD stage 4–5, end-stage renal failure or dialysis dependence.

In the spring-summer booster analysis, of the 182 SARS-CoV-2 cases, 78 (43%) received a fourth monovalent vaccine dose and 104 (57%) received a third monovalent vaccine dose, while 413 of 682 controls (61%) received a fourth monovalent vaccine dose and 269 (39%) received a third monovalent vaccine dose. All those vaccinated with a fourth monovalent dose were hospitalised ≤ 3 months after their vaccination and 98% of those who had received only three doses were hospitalised > 3 months after their vaccination; for further detail on admissions’ characteristics by vaccination group see Supplementary Table S2. The unadjusted rVE was 51.2% (95% confidence interval (CI): 32.1–65.0) and after adjustment, rVE was 46.6% (95% CI: 13.9–67.1). Matched conditional logistic regression rVE was 52% (95% CI: 20.9–70.9) (Table 3). A sensitivity analysis including individuals with three doses as primary vaccination regimen (who made up 8.5% of cases and 5.8% of controls) resulted in lower rVE estimates compared with results from the main analysis (Table 3); additional details on the sensitivity analysis and analytical results are appended in Supplementary Tables S4, S5 and S6.

**Table 3:** Relative vaccine effectiveness of fourth dose mRNA monovalent vaccines against hospitalisation, compared with third dose monovalent mRNA vaccines, 4 April–30 July 2022 (n = 864)

Characteristic	rVE (95% CI)	rOR (95% CI)	p value
Univariable logistic regression model			
Fourth dose of monovalent mRNA vaccine	51.2 (32.1–65.0)	0.488 (0.350–0.679)	< 0.001
Multivariable logistic regression model			
Fourth dose of monovalent mRNA vaccine	46.6 (13.9–67.1)	0.534 (0.329–0.861)	0.011
Time between third dose and admission	NA	1.004 (0.999–1.009)	0.13
Vaccine brand^a^		0.980 (0.636–1.509)	> 0.9
Age		1.010 (0.981–1.039)	0.5
Sex (male)		1.139 (0.803–1.615)	0.5
CCI		0.972 (0.873–1.077)	0.6
IMD		0.974 (0.914–1.037)	0.4
LTCF Resident		0.443 (0.221–0.819)	0.014
Respiratory disease		0.640 (0.440–0.926)	0.019
Prevalence^b^		1.001 (1.001–1.002)	< 0.001
Matched conditional logistic regression model^c^			
4th dose of monovalent mRNA vaccine	52.0 (20.9–70.9)	0.480 (0.291–0.791)	0.004
Time between third dose and admission	NA	1.005 (0.999–1.010)	0.088
Vaccine brand^a^		1.002 (0.634–1.584)	> 0.9
Prevalence^b^		1.002 (1.001–1.002)	< 0.001

CI: confidence interval; CCI: Charlson comorbidity index; LTCF: long-term care facility; IMD: index of multiple deprivation; NA: not applicable; rOR: relative odds ratio; rVE: relative vaccine effectiveness.

^a^ Vaccine brand of fourth dose of monovalent mRNA vaccine or third dose of monovalent mRNA vaccine (before last dose, any vaccine combination is considered), where 1 = SpikeVax and 0 = Comirnaty.

^b^ Prevalence was calculated on a daily basis.

^c^ 1:3 nearest neighbour propensity score matching with replacement (propensity score estimated using logistic regression on age, sex, CCI score, IMD, LTCF residency and respiratory disease), 179 test-positive cases were matched to 537 test-negative controls with no match found for 138 controls.

In the autumn-winter booster analysis, of the 152 SARS-CoV-2 cases, 100 (66%) received a fifth BA.1/ancestral bivalent vaccine dose and 52 (34%) received a fourth monovalent vaccine dose, while 572 of 732 (78%) controls received a fifth bivalent vaccine dose and 160 (22%) received a fourth monovalent vaccine dose. All those vaccinated with a fifth BA.1/ancestral bivalent vaccine dose were hospitalised ≤ 3 months after their vaccination and 97% of those who had received only three doses were hospitalised > 3 months after their vaccination; for further detail on admissions’ characteristics by vaccination group see Supplementary Table S3. The unadjusted rVE was 46.2% (95% CI: 21.1–63.0) and after adjustment, rVE was 46.7% (95% CI: 18.0–65.1). Matched conditional logistic regression rVE was 48.8% (95% CI: 19.8–67.3) (Table 4). The inclusion of individuals with three doses as primary vaccination regimen (who made up 3.8% of cases and 6.9% of controls) produced estimates comparable with results from the main analysis (Table 4); we append additional details on the sensitivity analysis and analytical results in Supplementary Tables S7, S8 and S9.

**Table 4:** Relative vaccine effectiveness of fifth dose BA.1/ancestral mRNA bivalent vaccines against hospitalisation, compared with fourth dose monovalent mRNA vaccines, 21 September 2022–23 January 2023 (n = 884)

Characteristic	rVE (95% CI)	rOR (95% CI)	*P*-value
Univariable logistic regression model			
Fifth dose of bivalent mRNA vaccine	46.2 (21.1–63.0)	0.538 (0.370–0.789)	0.001
Multivariable logistic regression model			
Fifth dose of monovalent mRNA vaccine	46.7 (18.0–65.1)	0.533 (0.349–0.820)	0.004
Time between 4th dose and admission	NA	1.000 (0.997–1.003)	> 0.9
Vaccine brand^a^		0.882 (0.611–1.283)	0.5
Age		1.006 (0.974–1.038)	0.7
Sex (male)		1.329 (0.925–1.910)	0.12
CCI		1.005 (0.895–1.123)	> 0.9
IMD		1.024 (0.961–1.092)	0.5
LTCF resident		0.953 (0.525–1.646)	0.9
Respiratory disease		0.807 (0.551–1.173)	0.3
Prevalence^b^		1.006 (0.998–1.013)	0.13
Matched conditional logistic regression model^c^			
5th dose of monovalent mRNA vaccine	48.8 (19.8–67.3)	0.512 (0.327–0.802)	0.003
Time between fourth dose and admission	NA	1.000 (0.997–1.003)	0.8
Vaccine brand^a^		0.909 (0.620–1.335)	0.6
Prevalence^b^		1.006 (0.999–1.014)	0.10

CI: confidence interval; CCI: Charlson comorbidity index; IMD: index of multiple deprivation; LTCF: long-term care facility; NA: not applicable; rOR: relative odds ratio; rVE: relative vaccine effectiveness.

^a^ Vaccine brand of fifth dose of BA.1/ancestral mRNA bivalent mRNA vaccine or fourth dose of monovalent mRNA vaccine (before last dose, any vaccine combination is considered), where 1 = SpikeVax and 0 = Comirnaty.

^b^ Prevalence was calculated on a daily basis.

^c^ 1:4 nearest neighbour propensity score matching with replacement (propensity score estimated using logistic regression on age, sex, CCI score, IMD, LTCF residency and respiratory disease), 150 test-positive cases were matched to 600 test-negative controls with no match found for 123 controls.

## Discussion

In this analysis, we considered the public health implications of monovalent and BA.1/ancestral bivalent vaccine implementation, focusing on people aged ≥ 75 years, a high-risk group which was a primary target for the UK vaccination programme. Although COVID-19 vaccines have been shown to be effective against severe COVID-19 disease [4,31,32], it has not been possible to compare the effectiveness of monovalent boosters directly with BA.1/ancestral bivalent booster doses of mRNA COVID-19 vaccines in defined populations, because bivalent formulations rapidly and entirely replaced monovalent formulations in the most recent booster programmes. In this ongoing prospective study, we undertook a sequential sub-analysis of the two vaccines given as boosters during two booster programmes in the same calendar year. It provided evidence that monovalent vaccine (original ‘wild-type’ Comirnaty and Spikevax) and BA.1/ancestral mRNA bivalent vaccine (original ‘wild-type’/Omicron BA.1 Cominarty and SpikeVax) within 3 months after vaccination provided similar additional protection as that afforded by waned previous doses against hospitalisation from Omicron SARS-CoV-2 sub-variants in older individuals. However, this needs to be interpreted with caution since we don’t have evidence about the effects of the different subvariants circulating during the distribution of the two vaccine formulations. The prevalence of different genomic variants of SARS-CoV-2 in England over time is available in Supplementary Figure S1.

Specifically, we estimated that a fourth monovalent mRNA vaccine dose within 3 months after vaccination was associated with a 46.6% (95% CI: 13.9–67.1) additional protection against hospitalisation compared with waned three doses, in individuals ≥ 75 years, during Omicron BA.2, BA.4, BA.5 lineage dominance. Within 3 months of receiving a fifth bivalent mRNA vaccine dose, it is estimated to provide 46.7% (95% CI: 18.0–65.1) additional protection against hospitalisation compared with waned four doses, in the ≥ 75 years age group, even when assessed during a period when heterologous variants were circulating since BA.5, BA.4.6, BQ.1 and CH1.1 lineages, the XBB recombinant lineage and its mutation XBB.1.5 accounted for most of the identified cases in England [22]. In Supplementary Figure S1, we provide the prevalence of different genomic variants of SARS-CoV-2 in England.

Although our results demonstrate that both vaccine formulations combined in these booster programmes had benefits when used as boosters, we have insufficient case numbers to draw conclusions about individual vaccine brands or directly compare them. Importantly, this analysis was restricted to individuals ≥ 75-years-old, with 97–98% of not fully boosted individuals in our sample potentially having waned vaccine-induced immunity since they received their last dose more than 3 months before admission. Given that this study had a short follow-up period after the administration of the two boosters, we cannot provide estimates by time since vaccination; nonetheless it presents encouraging evidence of similar benefit of monovalent and bivalent boosters in older adults, up to 3 months after vaccination. Older adults are at increased risk of severe disease, and protection may wane faster [33]; older adults were therefore targeted in the UK spring-summer 2022 and autumn-winter 2022 COVID-19 booster programmes. Our analysis based on the inclusion of individuals with severely weakened immune systems who were eligible for three primary doses had insufficient statistical power to draw firm conclusions, since they accounted for < 7% of our sample.

Vaccination against SARS-CoV-2 is independently associated with lower COVID-19 severity [4,31,32], and vaccines have been an important disease modifier during the pandemic. Although based on relatively small sample size, our estimates suggest that the bivalent boosters provided similar protection as monovalent boosters in a real-world setting where the landscape of COVID-19 variants is constantly changing: in small studies, results concordant with early evidence suggesting neutralising antibody titres induced against the Omicron variant by a bivalent booster dose were not higher than following a monovalent booster dose [16,17]. Our results are concordant with a UKHSA analysis [34] which estimated that the incremental protection conferred by a fourth monovalent dose compared with waned third dose was 58.8% (95% CI: 54.1–63.0), while the additional protection of BA.1/ancestral mRNA bivalent vaccines relative to those with two or more doses and waned protection was 43.1% (95% CI: 32.3–52.3) for Cominarty and 57.8% (95% CI: 51.2–63.5) for SpikeVax, during the same time period as our analysis.

Since the study took place over the course of two different time periods, the interpretation of these sequential analyses of the two vaccine formulations has to take into account the different variants circulating [22,29,30]. In England, the Omicron variants BA.2, BA.4, BA.5 were the main circulating variants during the study period of the monovalent booster and were replaced by BA.4 and BA.5 descendent sub-lineages (BA.4.6, BQ.1), CH1.1, XBB and XBB.1.5 lineage during the study period of the BA.1/ancestral bivalent boosters, with BA.5 being the only subvariant in common. Consequently, in this study, the performance of the BA.1/ancestral bivalent booster was not evaluated against homologous subvariants but against BA.4/5 which show further immune escape beyond that observed for BA.1. Currently, there is no evidence that Omicron BA.4-related sublineages, Omicron BA.5-related sublineages, CH1.1 and XBB recombinant-related sublineages, which appeared during the study period of the BA.1/ancestral mRNA bivalent boosters, cause more severe disease. The impact of these lineages on the effectiveness of the BA.1/ancestral mRNA bivalent formulation has not yet been studied in detail.

The test-negative design has been described previously, along with its advantages and limitations [12,25,35], and our analysis has some important additional strengths and limitations. The strength of our approach is the focus on using real-world data, while accounting for the potential effects of LTCF residency status, socioeconomic status and comorbidities. By limiting our analysis to boosted individuals only, our analysis sidesteps the potentially unfair comparisons between populations who have followed UK COVID-19 vaccine recommendations and unvaccinated populations who may display other idiosyncratic behaviours. We also used symptom onset date to define illness onset and were able to confirm that there was no difference in time since vaccination between the case and control groups compared. We therefore defined symptom onset relative to both vaccination and hospitalisation date accurately, without relying on positive test date (which may vary widely), eliminating this source of bias or misclassification. All patients were hospitalised with acute respiratory illness, so these results are unlikely to be subject to significant bias caused by admission for other causes (i.e. incidental COVID-19 disease). Most notably, our estimates for the effect of individual vaccines are underpowered, due to small patient numbers in our cohort during the phases of the UK vaccination programme and study periods. We were unable to assess additional protection against other markers of disease severity, such as admission to intensive care or requirement for respiratory support, due to the small number of eligible admissions in this time-period. This analysis did not measure rVE in individuals who were not hospitalised or were asymptomatic, so we cannot determine protection against asymptomatic disease or transmission. The cohort analysed here may have been subject to biases in treatment that we could not account for, for example, community-based treatment, death before admission or other reasons for non-referral to hospital, which may have resulted in a different cohort of hospitalised patients than that seen in other populations. We note that this cohort, while broadly representative of the UK population, was predominantly Caucasian and the studied vaccines may have different effectiveness in individuals from other ethnic backgrounds.

## Conclusion

In this prospective study, we provide evidence that autumn BA.1/ancestral mRNA bivalent COVID-19 boosters offered similar augmentation of protection against hospitalisation following infection with SARS-CoV-2 Omicron to that induced by spring monovalent mRNA boosters in 2022. These findings support immunisation programmes in the UK and several European countries that advised the use of BA.1/ancestral mRNA bivalent booster doses in high-risk individuals.

## Supplementary Data

547000 Supplement
