# Supplementary material for: Relative vaccine effectiveness of mRNA COVID-19 boosters in people aged at least 75 years during the spring-summer (monovalent vaccine) and autumn-winter (bivalent vaccine) booster campaigns: a prospective test negative case–control study, United Kingdom, 2022
Source: Euro Surveill. 2023 Nov 30;28(48):2300173. doi: 10.2807/1560-7917.ES.2023.28.48.2300173 (PMC10690860; doi:10.2807/1560-7917.ES.2023.28.48.2300173)
Supplement: Supplement [file 23-00173_DANON_Supplement.pdf]

SUPPLEMENTARY MATERIAL

This supplementary material is hosted by *Eurosurveillance* as supporting information alongside the article "Relative vaccine effectiveness of mRNA COVID-19 boosters in people aged at least 75 years during the spring-summer (monovalent vaccine) and autumn-winter (bivalent vaccine) booster campaigns: a prospective test negative case–control study, United Kingdom, 2022", on behalf of the authors, who remain responsible for the accuracy and appropriateness of the content. The same standards for ethics, copyright, attributions and permissions as for the article apply. Supplements are not edited by *Eurosurveillance* and the journal is not responsible for the maintenance of any links or email addresses provided therein.

Supplementary Figure S1: Admissions by vaccination status in each cohort by admission date – Admissions by vaccination status in each cohort by vaccination date - Prevalence of different genomic variants of SARS-CoV-2 over time

Spring-Summer monovalent booster analysis [4th dose – 3rd dose monovalent mRNA vaccines] admitted to hospital (04/04/22-30/07/22)

Autumn-Winter bivalent booster analysis [5th dose BA.1/ancestral mRNA bivalent - 4th dose monovalent vaccines] admitted to hospital (21/09/22-23/01/23)

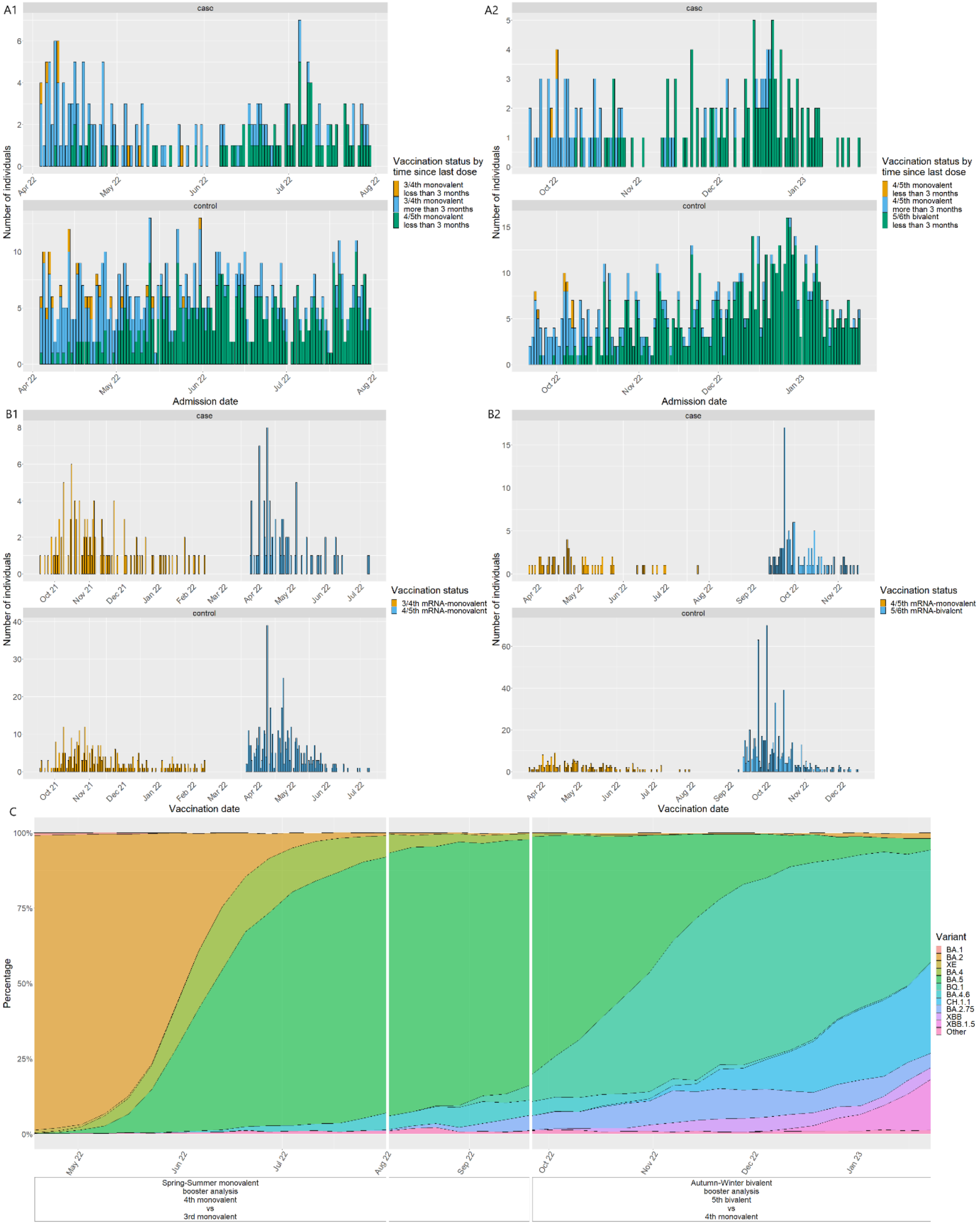

(A1,A2) The number of individuals in each cohort by vaccination status, by admission date. (B1,B2) The number of individuals in each cohort by vaccination status, by vaccination date. (C) Prevalence of different genomic variants of SARS-CoV-2 in available sequenced cases for England identified over time, as made available by UK Health Security Agency (UKHSA) (https://assets.publishing.service.gov.uk/government/uploads/system/uploads/attachment_data/file/1152143/variant-technical-briefing-52-21-april-2023.pdf)

Supplementary Table S1: Study design, inclusion and exclusion criteria

	**Spring-Summer monovalent booster analysis [4th dose – 3rd dose monovalent mRNA vaccines] admitted to hospital (04/04/22-30/07/22)**	**Autumn-Winter bivalent booster analysis [5th dose BA.1/ancestral mRNA bivalent - 4th dose monovalent vaccines] admitted to hospital (21/09/22-23/01/23)**
**Inclusion criteria**	• 4th dose mRNA-monovalent received after 21/03/22 and less than 3 months prior to admission<br>• 3rd dose mRNA-monovalent received between 16/09/21 and 14/02/22	• 5th dose mRNA-bivalent received after 07/09/22 and less than 3 months prior to admission<br>• 4th dose mRNA-monovalent received between 21/03/22 and 07/08/22
**Exclusion criteria**	• <2 symptoms, or not proven respiratory infection<br>• symptom onset >10 days before admission<br>• confirmed previous SARS-CoV-2 infection<br>• repeat admissions	• <2 symptoms, or not proven respiratory infection<br>• symptom onset >10 days before admission<br>• confirmed previous SARS-CoV-2 infection<br>• repeat admissions
	• <75 years old	• <75 years old
	• 4th dose mRNA-monovalent received before 21/03/22 (4th dose during Autumn-Winter 2021 booster programme i.e. they had 3 primary doses)<br>• 4th dose mRNA-monovalent received after 21/03/22 and more than 3 months prior to admission<br>• 4th dose ChAdOx1 (Vaxzevria®)<br>• 3rd dose mRNA-monovalent received before 15/09/21 or after 15/02/22<br>• 3rd dose ChAdOx1 (Vaxzevria®)<br>• Vaccinated with 5 (5th dose during Spring-Summer 2022 booster programme i.e. they had 3 primary doses), 2 doses or 1 dose<br>• Unvaccinated	• 5th dose mRNA-monovalent (5th dose during Spring-Summer 2022 booster programme i.e. they had 3 primary doses)5th dose mRNA-bivalent received after 07/09/22 and more than 3 months prior to admission<br>• 5th dose ChAdOx1 (Vaxzevria®)<br>• 4th dose mRNA-monovalent received before 20/03/22 or after 08/08/22<br>• 4th dose ChAdOx1 (Vaxzevria®)<br>• Vaccinated with 6 (6th dose during Autumn-Winter 2022 booster programme i.e. they had 3 primary doses), 2 doses or 1 dose<br>• Unvaccinated

Supplementary Table S2: Admission characteristics of study participants by vaccine type [4th dose – 3rd dose monovalent mRNA vaccines, ≥75 years admitted between 04/04/22 and 30/07/22]

Characteristic	4th mRNA-monovalent vaccine* (N = 491)	3rd mRNA-monovalent vaccine* (N = 373)	P-value
Cohort			<0.001
Case	78 (16%)	104 (28%)	
Control	413 (84%)	269 (72%)	
Vaccine brand#			<0.001
Moderna SpikeVax®	285 (58%)	39 (10%)	
Pfizer Comirnaty®	206 (42%)	334 (90%)	
Time since 3rd dose - median days (IQR)	224 (199, 249)	174 (156, 196)	<0.001
Time since 2nd dose - median days (IQR)	430 (406, 457)	381 (363, 411)	
Time since last dose - median days (IQR)	44 (22, 68)	174 (156, 196)	<0.001
Months since last dose			<0.001
≤ 3months	491 (100%)	7 (1.9%)	
> 3months	0 (0%)	366 (98%)	
Age - median years (IQR)	85 (80, 90)	84 (79, 89)	0.2
Sex			0.6
Male	232 (47%)	183 (49%)	
Female	259 (53%)	190 (51%)	
LTCF Resident	72 (15%)	34 (9.1%)	0.016
Ethnicity			>0.9
White British	387 (95%)	298 (95%)	
Other	22 (5.4%)	16 (5.1%)	
Unknown	82	59	
IMD – median (IQR)	5 (4, 8)	5 (4, 8)	0.5
Unknown	9	1	
Smoking			0.025
Current	17 (3.6%)	28 (7.9%)	
Ex-smoker	299 (64%)	209 (59%)	
Non-smoker	151 (32%)	117 (33%)	
Unknown	24	19	
Comorbidity scores			
Rockwood Frailty scale			0.7
1-4	92 (30%)	81 (32%)	
5-9	214 (70%)	175 (68%)	
Unknown	185	117	
CCI – median (IQR)	5 (4, 6)	5 (4, 7)	0.3
Respiratory			
Any	219 (45%)	150 (40%)	0.2
COPD	157 (32%)	110 (29%)	0.5
Asthma	51 (10%)	34 (9.1%)	0.6
Other⁑	51 (10%)	35 (9.4%)	0.6
Cardiovascular			
Any	284 (58%)	202 (54%)	0.3
IHD	90 (18%)	65 (17%)	0.8
AF	156 (32%)	121 (32%)	0.9
CCF	128 (26%)	93 (25%)	0.8
Diabetes			
Any	84 (17%)	104 (28%)	<0.001
Type 1	0	0	
Type 2	84 (100%)	104 (100%)	
Neurological			
Dementia	67 (14%)	50 (13%)	>0.9
Cognitive impairment	29 (5.9%)	23 (6.2%)	0.9
CVA	50 (10%)	41 (11%)	0.7

TIA	36 (7.3%)	45 (12%)	0.025
Other neurological disease†	20 (4.1%)	23 (6.2%)	0.2
Immunodeficiency			
CTD	51 (10%)	35 (9.4%)	0.6
HIV	0 (0%)	0 (0%)	
Other immunodeficiency	39 (7.9%)	28 (7.5%)	0.9
Oncology			
Solid organ cancer	53 (11%)	34 (9.1%)	0.4
Haematological malignancy	6 (1.2%)	4 (1.1%)	>0.9
Renal disease‡			0.3
None	305 (62%)	214 (57%)	
Mild	167 (34%)	138 (37%)	
Moderate/severe	19 (3.9%)	21 (5.6%)	

Data are N (%) unless otherwise stated.

*4th dose of monovalent mRNA vaccine after 21/03/22 and less than 3 months prior to admission.
3rd dose of monovalent mRNA vaccine between 16/09/21 and 14/02/22.

#Refers to vaccine brand of the last dose received (4th or 3rd dose). Prior to last dose, any vaccine combination is considered.
⁑Includes bronchiectasis, pulmonary fibrosis, and other chronic respiratory conditions.
†Includes Parkinson’s disease, Huntingdon’s disease, and other chronic neurological conditions.
‡Mild is CKD stage 1–3; moderate or severe is CKD stage 4–5, end-stage renal failure, or dialysis dependence.

AF, atrial fibrillation; CCF, congestive cardiac failure; CCI, Charlson Comorbidity Index; COPD, chronic obstructive pulmonary disease; CKD; chronic kidney disease; CVA, cerebrovascular accident; IHD, ischaemic heart disease; IMD, Index of multiple deprivation; IQR, interquartile range; LTCF, long-term care facility; TIA, transient ischaemic attack

Supplementary Table S3: Admission characteristics of study participants by vaccine type [5th dose BA.1/ancestral mRNA bivalent - 4th dose monovalent vaccines, ≥75 years admitted between 21/09/22 and 23/01/23]

Characteristic	5th mRNA-bivalent vaccine* (N = 672)	4th mRNA-monovalent vaccine * (N = 212)	P-value
Cohort			0.002
Case	100 (15%)	52 (25%)	
Control	572 (85%)	160 (75%)	
Vaccine brand[#]			0.003
Moderna SpikeVax®	455 (68%)	119 (56%)	
Pfizer Comirnaty®	217 (32%)	93 (44%)	
Time since 4th dose - median days (IQR)	223 (194, 250)	166 (147, 190)	<0.001
Time since 3rd dose - median days (IQR)	402 (376, 425)	350 (333, 377)	<0.001
Time since 2nd dose - median days (IQR)	609 (582, 628)	553 (532, 597)	<0.001
Time since last dose - median days (IQR)	59 (32, 76)	166 (147, 190)	<0.001
Months since last dose			<0.001
≤ 3months	672 (100%)	7 (3.3%)	
> 3months	0 (0%)	205 (97%)	
Age - median years (IQR)	84 (80, 89)	86 (81, 89)	0.15
Sex			0.8
Male	304 (45%)	99 (47%)	
Female	368 (55%)	113 (53%)	
LTCF Resident	81 (12%)	24 (11%)	>0.9
Ethnicity			0.3
White British	529 (98%)	163 (96%)	
Other	11 (2.0%)	6 (3.6%)	
Unknown	132	43	
IMD – median (IQR)	6 (4, 9)	6 (4, 9)	0.5
Unknown	9	2	
Smoking			0.7
Current	40 (6.3%)	10 (4.9%)	
Ex-smoker	409 (65%)	129 (63%)	
Non-smoker	184 (29%)	65 (32%)	
Unknown	39	8	
Comorbidity scores			
Rockwood Frailty scale			0.11
1-4	246 (46%)	68 (39%)	
5-9	286 (54%)	105 (61%)	
Unknown	140	39	
CCI – median (IQR)	5 (4, 6)	5 (5, 6)	0.2
Respiratory			
Any	285 (42%)	96 (45%)	0.5
COPD	185 (28%)	71 (33%)	0.10
Asthma	87 (13%)	20 (9.4%)	0.2
Other⁑	61 (9.1%)	21 (9.9%)	0.7
Cardiovascular			
Any	365 (54%)	128 (60%)	0.13
IHD	105 (16%)	36 (17%)	0.7
AF	205 (31%)	84 (40%)	0.015
CCF	159 (24%)	51 (24%)	>0.9
Diabetes			
Any	120 (18%)	45 (21%)	0.3
Type 1	1 (0.8%)	0 (0%)	>0.9
Type 2	119 (99%)	45 (100%)	
Neurological			
Dementia	83 (12%)	24 (11%)	0.8

Cognitive impairment	50 (7.4%)	24 (11%)	0.087
CVA	59 (8.8%)	26 (12%)	0.14
TIA	62 (9.2%)	20 (9.4%)	0.9
Other neurological disease†	33 (4.9%)	12 (5.7%)	0.7
Immunodeficiency			
CTD	63 (9.4%)	16 (7.5%)	0.5
HIV	0 (0%)	1 (0.5%)	0.2
Other immunodeficiency	99 (15%)	24 (11%)	0.3
Oncology			
Solid organ cancer	60 (8.9%)	17 (8.0%)	0.8
Haematological malignancy	21 (3.1%)	4 (1.9%)	0.5
Renal disease‡			0.5
None	353 (53%)	121 (57%)	
Mild	287 (43%)	84 (40%)	
Moderate/severe	32 (4.8%)	7 (3.3%)	

Data are N (%) unless otherwise stated.

*5th dose of BA.1/ancestral mRNA bivalent mRNA vaccine after 07/09/22 and less than 3 months prior to admission
4th dose of monovalent mRNA vaccine between 21/03/22 and 07/08/22.

#Refers to vaccine brand of the last dose received (5th or 4th dose). Prior to last dose, any vaccine combination is considered.
⁑Includes bronchiectasis, pulmonary fibrosis, and other chronic respiratory conditions.
†Includes Parkinson's disease, Huntingdon's disease, and other chronic neurological conditions.
‡Mild is CKD stage 1–3; moderate or severe is CKD stage 4–5, end-stage renal failure, or dialysis dependence.

AF, atrial fibrillation; CCF, congestive cardiac failure; CCI, Charlson Comorbidity Index; COPD, chronic obstructive pulmonary disease; CKD; chronic kidney disease; CVA, cerebrovascular accident; IHD, ischaemic heart disease; IMD, Index of multiple deprivation; IQR, interquartile range; LTCF, long-term care facility; TIA, transient ischaemic attack

Supplementary Table S4: Admission characteristics of study participants [4/5th dose – 3/4th dose monovalent mRNA vaccines, ≥75 years admitted between 04/04/22 and 30/07/22, including vaccinated with 3 primary doses]

Characteristic	Cases SARS-CoV-2 positive (N = 199)	Controls SARS-CoV-2 negative (N = 724)	*P*-value
Vaccination status*			<0.001
4/5th mRNA-monovalent	83 (42%)	419 (58%)	
3/4th mRNA-monovalent	116 (58%)	305 (42%)	
Vaccine brand#			0.011
Moderna SpikeVax®	62 (31%)	298 (41%)	
Pfizer Comirnaty®	137 (69%)	426 (59%)	
Time since 3/4th dose - median days (IQR)	187 (160, 230)	202 (168, 235)	0.011
Time since 2/3rd dose - median days (IQR)	390 (361, 441)	409 (378, 442)	<0.001
Time since last dose - median days (IQR)	135 (65, 174)	74 (37, 163)	<0.001
Months since last dose			<0.001
≤ 3months	90 (45%)	441 (61%)	
> 3months	109 (55%)	283 (39%)	
Primary vaccination regime			0.2
2-dose	182 (91%)	682 (94%)	
3-dose	17 (8.5%)	42 (5.8%)	
Age - median years (IQR)	84 (79, 90)	84 (79, 89)	0.6
Sex			0.3
Male	106 (53%)	351 (48%)	
Female	93 (47%)	373 (52%)	
LTCF Resident	13 (6.5%)	94 (13%)	0.012
Ethnicity			0.029
White British	161 (91%)	575 (96%)	
Other	15 (8.5%)	24 (4.0%)	
Unknown	23	125	
IMD – median (IQR)	5 (4, 8)	6 (4, 8)	>0.9
Unknown	3	7	
Smoking			0.08
Current	13 (6.7%)	34 (5.0%)	
Ex-smoker	106 (55%)	437 (64%)	
Non-smoker	74 (38%)	215 (31%)	
Unknown	6	38	
Comorbidity scores			
Rockwood Frailty scale			0.3
1-4	50 (35%)	138 (30%)	
5-9	91 (65%)	315 (70%)	
Unknown	58	271	
CCI – median (IQR)	5 (4, 6)	5 (5, 6)	>0.9
Respiratory			
Any	74 (37%)	335 (46%)	0.024
COPD	47 (24%)	246 (34%)	0.006
Asthma	19 (9.5%)	76 (10%)	0.8
Other‡	22 (11%)	84 (12%)	>0.9
Cardiovascular			
Any	108 (54%)	413 (57%)	0.5
IHD	32 (16%)	130 (18%)	0.6
AF	63 (32%)	233 (32%)	>0.9
CCF	47 (24%)	187 (26%)	0.6
Diabetes			
Any	47 (24%)	155 (21%)	0.5
Type 1	0 (0%)	1 (0.6%)	>0.9
Type 2	47 (100%)	154 (99%)	
Neurological			
Dementia	24 (12%)	95 (13%)	0.8

Cognitive impairment	15 (7.5%)	38 (5.2%)	0.2
CVA	18 (9.0%)	76 (10%)	0.6
TIA	15 (7.5%)	73 (10%)	0.3
Other neurological disease†	12 (6.0%)	34 (4.7%)	0.5
Immunodeficiency			
CTD	24 (12%)	75 (10%)	0.5
HIV	1 (0.5%)	0 (0%)	0.2
Other immunodeficiency	19 (9.5%)	60 (8.3%)	0.6
Oncology			
Solid organ cancer	20 (10%)	75 (10%)	>0.9
Haematological malignancy	7 (3.5%)	14 (1.9%)	0.2
Renal disease‡			0.5
None	117 (59%)	433 (60%)	
Mild	69 (35%)	258 (36%)	
Moderate/severe	13 (6.5%)	33 (4.6%)	

Data are N (%) unless otherwise stated.

*Vaccinated individuals who have received a 4/5th dose of monovalent mRNA vaccine after 21/03/22 and less than 3 months prior to admission or a 3/4th dose of monovalent mRNA vaccine between 16/09/21 and 14/02/22.

#Refers to vaccine brand of the last dose received (4/5th or 3/4th dose). Prior to last dose, any vaccine combination is considered.

⁑Includes bronchiectasis, pulmonary fibrosis, and other chronic respiratory conditions.
†Includes Parkinson's disease, Huntingdon's disease, and other chronic neurological conditions.
‡Mild is CKD stage 1–3; moderate or severe is CKD stage 4–5, end-stage renal failure, or dialysis dependence.

AF, atrial fibrillation; CCF, congestive cardiac failure; CCI, Charlson Comorbidity Index; COPD, chronic obstructive pulmonary disease; CKD; chronic kidney disease; CVA, cerebrovascular accident; IHD, ischaemic heart disease; IMD, Index of multiple deprivation; IQR, interquartile range; LTCF, long-term care facility; TIA, transient ischaemic attack

Supplementary Table S5: Admission characteristics of study participants by vaccine type [4/5th dose – 3/4th dose monovalent mRNA vaccines, ≥75 years admitted between 04/04/22 and 30/07/22, including vaccinated with 3 primary doses]

Characteristic	4/5th mRNA-monovalent vaccine* (N = 502)	3/4th mRNA-monovalent vaccine* (N = 421)	P-value
Cohort			<0.001
Case	83 (17%)	116 (28%)	
Control	419 (83%)	305 (72%)	
Vaccine brand#			<0.001
Moderna SpikeVax®	292 (58%)	68 (16%)	
Pfizer Comirnaty®	210 (42%)	353 (84%)	
Time since 3/4th dose - median days (IQR)	224 (197, 247)	170 (148, 192)	<0.001
Time since 2/3th dose - median days (IQR)	430 (404, 457)	376 (353, 405)	<0.001
Time since last dose - median days (IQR)	44 (21, 68)	170 (148, 192)	<0.001
Months since last dose			<0.001
≤ 3months	502 (100%)	29 (6.9%)	
> 3months	0 (0%)	392 (93%)	
Primary vaccination regime			<0.001
2-dose	491 (98%)	373 (89%)	
3-dose	11 (2.2%)	48 (11%)	
Age - median years (IQR)	85 (80, 90)	83 (79, 88)	0.044
Sex			0.3
Male	240 (48%)	217 (52%)	
Female	262 (52%)	204 (48%)	
LTCF Resident	72 (14%)	35 (8.3%)	0.005
Ethnicity			0.9
White British	396 (95%)	340 (95%)	
Other	22 (5.3%)	17 (4.8%)	
Unknown	84	64	
IMD – median (IQR)	5 (4, 8)	6 (4, 9)	0.2
Unknown	9	1	
Smoking			0.027
Current	17 (3.6%)	30 (7.5%)	
Ex-smoker	307 (64%)	236 (59%)	
Non-smoker	154 (32%)	135 (34%)	
Unknown	24	20	
Comorbidity scores			
Rockwood Frailty scale			0.5
1-4	95 (30%)	93 (33%)	
5-9	218 (70%)	188 (67%)	
Unknown	189	140	
CCI – median (IQR)	5 (5, 6)	5 (4, 7)	0.2
Respiratory			
Any	227 (45%)	182 (43%)	0.6
COPD	160 (32%)	133 (32%)	>0.9
Asthma	54 (11%)	41 (9.7%)	0.7
Other⁑	56 (11%)	50 (12%)	0.8
Cardiovascular			
Any	292 (58%)	229 (54%)	0.3
IHD	90 (18%)	72 (17%)	0.8
AF	159 (32%)	137 (33%)	0.8
CCF	134 (27%)	100 (24%)	0.3
Diabetes			
Any	86 (17%)	116 (28%)	<0.001
Type 1	0 (0%)	1 (0.9%)	>0.9
Type 2	86 (100%)	115 (99%)	
Neurological			

Dementia	67 (13%)	52 (12%)	0.7
Cognitive impairment	29 (5.8%)	24 (5.7%)	>0.9
CVA	51 (10%)	43 (10%)	>0.9
TIA	38 (7.6%)	50 (12%)	0.032
Other neurological disease†	20 (4.0%)	26 (6.2%)	0.13
Immunodeficiency			
CTD	52 (10%)	47 (11%)	0.7
HIV	0 (0%)	1 (0.2%)	0.5
Other immunodeficiency	40 (8.0%)	39 (9.3%)	0.6
Oncology			
Solid organ cancer	54 (11%)	41 (9.7%)	0.7
Haematological malignancy	8 (1.6%)	13 (3.1%)	0.2
Renal disease‡			0.13
None	312 (62%)	238 (57%)	
Mild	170 (34%)	157 (37%)	
Moderate/severe	20 (4.0%)	26 (6.2%)	

Data are N (%) unless otherwise stated.

*4/5th dose of monovalent mRNA vaccine after 21/03/22 and less than 3 months prior to admission.
3/4th dose of monovalent mRNA vaccine between 16/09/21 and 14/02/22.

#Refers to vaccine brand of the last dose received (4/5th or 3/4th dose). Prior to last dose, any vaccine combination is considered.

⁑Includes bronchiectasis, pulmonary fibrosis, and other chronic respiratory conditions.
†Includes Parkinson's disease, Huntingdon's disease, and other chronic neurological conditions.
‡Mild is CKD stage 1–3; moderate or severe is CKD stage 4–5, end-stage renal failure, or dialysis dependence.

AF, atrial fibrillation; CCF, congestive cardiac failure; CCI, Charlson Comorbidity Index; COPD, chronic obstructive pulmonary disease; CKD; chronic kidney disease; CVA, cerebrovascular accident; IHD, ischaemic heart disease; IMD, Index of multiple deprivation; IQR, interquartile range; LTCF, long-term care facility; TIA, transient ischaemic attack

Supplementary Table S6: Relative vaccine effectiveness of 4/5th dose mRNA monovalent vaccines against hospitalisation, compared to 3/4th dose monovalent mRNA vaccines [04/04/22-30/07/22, including vaccinated with 3 primary doses]

Characteristic	rVE (95% CI)	rOR (95% CI)	*P*-value
**Univariable Logistic Regression Model**			
4/5th dose of monovalent mRNA vaccine	47.9 (28.5, 62.2)	0.521 (0.378, 0.715)	<0.001
**Multivariable Logistic Regression Model**			
4/5th dose of monovalent mRNA vaccine	37.4 (2.1, 60.2)	0.626 (0.398, 0.979)	0.041
Time between 3/4th dose and admission		1.004 (0.999, 1.008)	0.14
Vaccine brand*		0.878 (0.587, 1.310)	0.5
Primary vaccination series⁑		0.508 (0.248, 1.063)	0.066
Age		1.008 (0.980, 1.037)	0.6
Sex (Male)		1.057 (0.756, 1.479)	0.7
CCI		0.974 (0.879, 1.074)	0.6
IMD		0.969 (0.912, 1.030)	0.3
LTCF Resident		0.434 (0.217, 0.799)	0.011
Respiratory disease		0.643 (0.451, 0.913)	0.014
Prevalence#		1.001 (1.001, 1.002)	<0.001
**Matched Conditional Logistic Regression Model†**			
4/5th dose of monovalent mRNA vaccine	37.9 (1.0, 61.0)	0.621 (0.390, 0.990)	0.045
Time between 3/4th dose and admission		1.004 (0.999, 1.008)	0.12
Vaccine brand		0.820 (0.548, 1.227)	0.3
Primary vaccination series⁑		0.583 (0.284, 1.199)	0.14
Prevalence#		1.001 (1.001, 1.002)	<0.001

CI, Confidence Interval; CCI, Charlson comorbidity index; IMD, index of multiple deprivation; rOR, relative Odds Ratio; rVE, relative vaccine effectiveness

* Vaccine brand of 4/5th dose of monovalent mRNA vaccine or 3/4th dose of monovalent mRNA vaccine (prior to last dose, any vaccine combination is considered), where 1= Moderna SpikeVax® and 0 = Pfizer Comirnaty®

⁑ Primary vaccination series, where 1= 2 doses before autumn-winter 2021 and 0= 3 doses before autumn-winter 2021

#Prevalence was calculated on a daily basis

†1:3 Nearest neighbour propensity score matching with replacement (propensity score estimated using logistic regression on age, sex, CCI score, IMD, LTCF residency and respiratory disease), 196 test-positive cases were matched to 588 test-negative controls with no match found for 129 controls.

Supplementary Table S7: Admission characteristics of study participants [5/6th dose BA.1/ancestral mRNA bivalent – 4/5th dose monovalent vaccines, ≥75 years admitted between 21/09/22 and 23/01/23, including vaccinated with 3 primary doses]

Characteristic	Cases SARS-CoV-2 positive (N = 158)	Controls SARS-CoV-2 negative (N = 786)	*P*-value
Vaccination status*			0.002
5th mRNA-bivalent	103 (65%)	610 (78%)	
4th mRNA-monovalent	55 (35%)	176 (22%)	
Vaccine brand#			0.2
Moderna SpikeVax®	95 (60%)	517 (66%)	
Pfizer Comirnaty®	63 (40%)	269 (34%)	
Time since 4/5th dose - median days (IQR)	204 (164, 237)	207 (176, 242)	0.3
Time since 3/4th dose - median days (IQR)	384 (346, 418)	390 (355, 419)	0.6
Time since 2/3rd dose - median days (IQR)	592 (548, 620)	597 (560, 623)	0.5
Time since last dose - median days (IQR)	75 (52, 144)	67 (40, 97)	0.03
Months since last dose			0.001
≤ 3months	105 (66%)	618 (79%)	
> 3months	53 (34%)	168 (21%)	
Primary vaccination regime			0.2
2-dose	152 (96%)	732 (93%)	
3-dose	6 (3.8%)	54 (6.9%)	
Age - median years (IQR)	84 (80, 89)	85 (80, 89)	0.4
Sex			0.2
Male	80 (51%)	351 (45%)	
Female	78 (49%)	435 (55%)	
LTCF Resident	19 (12%)	89 (11%)	0.8
Ethnicity			0.3
White British	113 (97%)	627 (98%)	
Other	4 (3.4%)	13 (2.0%)	
Unknown	41	146	
IMD – median (IQR)	6 (4, 9)	6 (4, 9)	0.6
Unknown	2	10	
Smoking			0.005
Current	2 (1.4%)	53 (7.1%)	
Ex-smoker	89 (61%)	482 (64%)	
Non-smoker	55 (38%)	215 (29%)	
Unknown	12	36	
Comorbidity scores			
Rockwood Frailty scale			0.14
1-4	47 (39%)	290 (46%)	
5-9	75 (61%)	336 (54%)	
Unknown	36	160	
CCI – median (IQR)	5 (4, 6)	5 (4, 7)	0.7
Respiratory			
Any	61 (39%)	346 (44%)	0.2
COPD	39 (25%)	237 (30%)	0.2
Asthma	18 (11%)	94 (12%)	>0.9
Other⁑	10 (6.3%)	79 (10%)	0.2
Cardiovascular			
Any	87 (55%)	440 (56%)	0.9
IHD	31 (20%)	123 (16%)	0.2
AF	50 (32%)	258 (33%)	0.9
CCF	31 (20%)	190 (24%)	0.3
Diabetes			
Any	26 (16%)	157 (20%)	0.4
Type 1	0 (0%)	1 (0.6%)	>0.9

Type 2	26 (100%)	156 (99%)	
Neurological			
Dementia	27 (17%)	83 (11%)	0.029
Cognitive impairment	18 (11%)	58 (7.4%)	0.11
CVA	21 (13%)	69 (8.8%)	0.10
TIA	18 (11%)	66 (8.4%)	0.2
Other neurological disease†	11 (7.0%)	36 (4.6%)	0.2
Immunodeficiency			
CTD	18 (11%)	76 (9.7%)	0.6
HIV	0 (0%)	1 (0.1%)	>0.9
Other immunodeficiency	23 (15%)	120 (15%)	>0.9
Oncology			
Solid organ cancer	12 (7.6%)	77 (9.8%)	0.5
Haematological malignancy	4 (2.5%)	32 (4.1%)	0.5
Renal disease‡			0.5
None	91 (58%)	417 (53%)	
Mild	62 (39%)	330 (42%)	
Moderate/severe	5 (3.2%)	39 (5.0%)	

Data are N (%) unless otherwise stated.

*Vaccinated individuals who have received a 5/6th dose of BA.1/ancestral mRNA bivalent mRNA vaccine after 07/09/22 and less than 3 months prior to admission or a 4/5th dose of monovalent mRNA vaccine between 21/03/22 and 07/08/22.

#Refers to vaccine brand of the last dose received (5/6th or 4/5th dose). Prior to last dose, any vaccine combination is considered.

⁑Includes bronchiectasis, pulmonary fibrosis, and other chronic respiratory conditions.
†Includes Parkinson's disease, Huntingdon's disease, and other chronic neurological conditions.
‡Mild is CKD stage 1–3; moderate or severe is CKD stage 4–5, end-stage renal failure, or dialysis dependence.

AF, atrial fibrillation; CCF, congestive cardiac failure; CCI, Charlson Comorbidity Index; COPD, chronic obstructive pulmonary disease; CKD; chronic kidney disease; CVA, cerebrovascular accident; IHD, ischaemic heart disease; IMD, Index of multiple deprivation; IQR, interquartile range; LTCF, long-term care facility; TIA, transient ischaemic attack

Supplementary Table S8: Admission characteristics of study participants by vaccine type [5/6th dose BA.1/ancestral mRNA bivalent – 4/5th dose monovalent vaccines, ≥75 years admitted between 21/09/22 and 23/01/23, including vaccinated with 3 primary doses]

Characteristic	5/6th mRNA-bivalent vaccine* (N = 713)	4/5th mRNA-monovalent vaccine * (N = 231)	P-value
Cohort			0.002
Case	103 (14%)	55 (24%)	
Control	610 (86%)	176 (76%)	
Vaccine brand[#]			0.022
Moderna SpikeVax®	477 (67%)	135 (58%)	
Pfizer Comirnaty®	236 (33%)	96 (42%)	
Time since 4/5th dose - median days (IQR)	219 (191, 249)	164 (144, 188)	<0.001
Time since 3/4th dose - median days (IQR)	400 (371, 424)	346 (327, 373)	<0.001
Time since 2/3rd dose - median days (IQR)	606 (577, 627)	549 (527, 590)	<0.001
Time since last dose - median days (IQR)	58 (32, 76)	164 (144, 188)	<0.001
Months since last dose			<0.001
≤ 3months	713 (100%)	10 (4.3%)	
> 3months	0 (0%)	221 (96%)	
Primary vaccination regime			0.2
2-dose	672 (94%)	212 (92%)	
3-dose	41 (5.8%)	19 (8.2%)	
Age – median years (IQR)	84 (80, 89)	85 (80, 89)	0.3
Sex			0.8
Male	324 (45%)	107 (46%)	
Female	389 (55%)	124 (54%)	
LTCF Resident	83 (12%)	25 (11%)	0.8
Ethnicity			0.4
White British	560 (98%)	180 (97%)	
Other	11 (1.9%)	6 (3.2%)	
Unknown	142	45	
IMD – median (IQR)	6 (4, 9)	6 (4, 9)	0.5
Unknown	10	2	
Smoking			0.3
Current	44 (6.5%)	11 (4.9%)	
Ex-smoker	434 (64%)	137 (61%)	
Non-smoker	195 (29%)	75 (34%)	
Unknown	40	8	
Comorbidity scores			
Rockwood Frailty scale			0.13
1-4	263 (47%)	74 (40%)	
5-9	300 (53%)	111 (60%)	
Unknown	150	46	
CCI – median (IQR)	5 (4, 6)	5 (5, 6)	0.3
Respiratory			
Any	303 (42%)	104 (45%)	0.5
COPD	200 (28%)	76 (33%)	0.2
Asthma	91 (13%)	21 (9.1%)	0.2
Other⁑	64 (9.0%)	25 (11%)	0.4
Cardiovascular			
Any	388 (54%)	139 (60%)	0.13
IHD	114 (16%)	40 (17%)	0.7
AF	220 (31%)	88 (38%)	0.044
CCF	168 (24%)	53 (23%)	>0.9
Diabetes			
Any	134 (19%)	49 (21%)	0.4
Type 1	1 (0.7%)	0 (0%)	>0.9

Type 2	133 (99%)	49 (100%)	
Neurological			
Dementia	86 (12%)	24 (10%)	0.6
Cognitive impairment	51 (7.2%)	25 (11%)	0.094
CVA	64 (9.0%)	26 (11%)	0.3
TIA	64 (9.0%)	20 (8.7%)	>0.9
Other neurological disease†	35 (4.9%)	12 (5.2%)	0.9
Immunodeficiency			
CTD	71 (10.0%)	23 (10.0%)	>0.9
HIV	0 (0%)	1 (0.4%)	0.2
Other immunodeficiency	110 (15%)	33 (14%)	0.8
Oncology			
Solid organ cancer	68 (9.5%)	21 (9.1%)	0.9
Haematological malignancy	28 (3.9%)	8 (3.5%)	0.8
Renal disease‡			0.3
None	374 (52%)	134 (58%)	
Mild	303 (42%)	89 (39%)	
Moderate/severe	36 (5.0%)	8 (3.5%)	

Data are N (%) unless otherwise stated.

*5/6th dose of BA.1/ancestral mRNA bivalent mRNA vaccine after 07/09/22 and less than 3 months prior to admission 4/5th dose of monovalent mRNA vaccine between 21/03/22 and 07/08/22.

#Refers to vaccine brand of the last dose received (5/6th or 4/5th dose). Prior to last dose, any vaccine combination is considered.

⁑Includes bronchiectasis, pulmonary fibrosis, and other chronic respiratory conditions.
†Includes Parkinson's disease, Huntingdon's disease, and other chronic neurological conditions.
‡Mild is CKD stage 1–3; moderate or severe is CKD stage 4–5, end-stage renal failure, or dialysis dependence.

AF, atrial fibrillation; CCF, congestive cardiac failure; CCI, Charlson Comorbidity Index; COPD, chronic obstructive pulmonary disease; CKD; chronic kidney disease; CVA, cerebrovascular accident; IHD, ischaemic heart disease; IMD, Index of multiple deprivation; IQR, interquartile range; LTCF, long-term care facility; TIA, transient ischaemic attack

Supplementary Table S9: Relative vaccine effectiveness of 5/6th dose BA.1/ancestral mRNA bivalent vaccines against hospitalisation, compared to 4/5th dose monovalent mRNA vaccines [21/09/22 – 23/01/23, including vaccinated with 3 primary doses]

Characteristic	rVE (95% CI)	rOR (95% CI)	*P*-value
**Univariable Logistic Regression Model**			
5/6th dose of monovalent mRNA vaccine	46.0 (21.6, 62.5)	0.540 (0.375, 0.784)	0.001
**Multivariable Logistic Regression Model**			
5/6th dose of monovalent mRNA vaccine	46.8 (18.9, 64.9)	0.532 (0.351, 0.811)	0.003
Time between 4/5th dose and admission		1.000 (0.996, 1.002)	0.8
Vaccine brand*		0.828 (0.579, 1.192)	0.3
Primary vaccination series⁑		1.963 (0.864, 5.307)	0.14
Age		1.004 (0.973, 1.036)	0.8
Sex (Male)		1.302 (0.913, 1.858)	0.14
CCI		0.981 (0.875, 1.093)	0.7
IMD		1.013 (0.952, 1.078)	0.7
LTCF Resident		1.004 (0.561 1.715)	>0.9
Respiratory disease		0.794 (0.546, 1.146)	0.2
Prevalence		1.006 (0.999, 1.014)	0.086
**Matched Conditional Logistic Regression Model†**			
5/6th dose of monovalent mRNA vaccine	44.9 (15.8, 64.0)	0.551 (0.360, 0.842)	0.006
Time between 4/5th dose and admission		1.000 (0.997, 1.003)	>0.9
Vaccine brand*		0.852 (0.591, 1.229)	0.4
Primary vaccination series⁑		1.970 (0.780, 4.976)	0.2
Prevalence#		1.007 (0.999, 1.014)	0.089

CI, Confidence Interval; CCI, Charlson comorbidity index; IMD, index of multiple deprivation; rOR, relative Odds Ratio; rVE, relative vaccine effectiveness

* Vaccine brand of 5/6th dose of BA.1/ancestral mRNA bivalent mRNA vaccine or 4/5th dose of monovalent mRNA vaccine (prior to last dose, any vaccine combination is considered), where 1= Moderna SpikeVax® and 0 = Pfizer Comirnaty®

⁑ Primary vaccination series, where 1= 2 doses before autumn-winter 2021 and 0= 3 doses before autumn-winter 2021

#Prevalence was calculated on a daily basis

†1:4 Nearest neighbour propensity score matching with replacement (propensity score estimated using logistic regression on age, sex, CCI score, IMD, LTCF residency and respiratory disease), 156 test-positive cases were matched to 624 test-negative controls with no match found for 152 controls.
